# Data Driven Models of Short-Term Synaptic Plasticity

**DOI:** 10.3389/fncom.2018.00032

**Published:** 2018-05-22

**Authors:** Elham Bayat Mokhtari, J. Josh Lawrence, Emily F. Stone

**Affiliations:** ^1^Department of Mathematical Sciences, The University of Montana, Missoula, MT, United States; ^2^Pharmacology and Neuroscience, Texas Tech University Health Sciences Center, Lubbock, TX, United States

**Keywords:** short term plasticity, epsilon machines, synaptic filtering, mutual information, interneuron-pyramidal cell synapses, causal state splitting reconstruction

## Abstract

Simple models of short term synaptic plasticity that incorporate facilitation and/or depression have been created in abundance for different synapse types and circumstances. The analysis of these models has included computing mutual information between a stochastic input spike train and some sort of representation of the postsynaptic response. While this approach has proven useful in many contexts, for the purpose of determining the type of process underlying a stochastic output train, it ignores the ordering of the responses, leaving an important characterizing feature on the table. In this paper we use a broader class of information measures on output only, and specifically construct hidden Markov models (HMMs) (known as epsilon machines or causal state models) to differentiate between synapse type, and classify the complexity of the process. We find that the machines allow us to differentiate between processes in a way not possible by considering distributions alone. We are also able to understand these differences in terms of the dynamics of the model used to create the output response, bringing the analysis full circle. Hence this technique provides a complimentary description of the synaptic filtering process, and potentially expands the interpretation of future experimental results.

## 1. Introduction

Short term plasticity at the synapse level can have profound effects on functional connectivity of neurons. Through repetitive activation, the strength, or efficacy, of synaptic release of neurotransmitter can be decreased, through depletion, or increased, through facilitation. A single synapse type can display different properties at different frequencies of stimulation.

The role of synaptic plasticity and computation has been analyzed and reported on in numerous papers over the past 30 years. A review of feed-forward synaptic mechanisms and their implications can be found in Abbott and Regehr (2004). In this paper Abbott and Regher state “The potential computational power of synapses is large because their basic signal transmission properties can be affected by the history of presynaptic and postsynaptic firing in so many different ways.” They also outline the basic function of a synapse as a signal filter as follows: Synapses with an initial low probability of release act as high pass filters through facilitation, while synapses with an initially high probability of release exhibit depression and subsequently serve as low pass filters. Intermediate cases in which the synapse can act as a band-pass filter, exist. Identifying synapse-specific molecular mechanisms is currently an active area of research, involving subtle changes in expression of myriad calcium sensor isoforms (synaptotagmins), subtly configured to alter the microscopic rates of synaptic release, facilitation, depression, and vesicle replenishment (Fioravante and Regehr, 2011; Chen and Jonas, 2017; Jackman and Regehr, 2017).

The underlying mechanisms creating these effects may be inferred by fitting an *a priori* model to synaptic response data. We parameterize such a model combining the properties of facilitation and depression (FD) at the presynaptic neuron with experimental data from dual whole-cell recordings from a presynaptic parvalbumin-positive (PV) basket cell (BC) connected to a postsynaptic CA1 (Cornu Ammonis 1 subregion) pyramidal cell, for fixed frequency spike trains into the presynaptic PV BC (Stone et al., 2014; Lawrence et al., 2015). We later examine the response of the model to an *in vivo*-like Poisson spike train of input, where the inter-spike interval (ISI) follows an exponential distribution, in Bayat et al. (submitted). Here we investigate the information processing properties of the synapse in question, following (Markram et al., 1998) and using standard calculations of entropy and mutual information between the input spike train and output response. This, however, left us unsatisfied, as it did not indicate the *history* dependence of the response, which we believe is one of the more interesting features of plasticity models that involve presynaptic calcium concentration. We attempted using multivariate mutual information measures, but this very quickly collapses due to the “curse of dimensionality” (Bellman, 1957). In this paper we try to resolve the question using methods of Computational Mechanics, creating unifilar HMMs called *epsilon machines*, that represent the stochastic process that our synapse model creates. As a bonus we are using data itself (albeit synthetic data) to create models of plasticity that can be used to classify properties of different types of synapses.

As stated in the abstract, methods from Information theory rely on distribution measures which inherently ignore the ordering of the measured data stream. We seek to incorporate this important feature of plasticity, the dependence of the response of the synapse on the prior sequence of stimulation, directly through the construction of causal state machines. This can only add to the understanding of the process in cases where the input stimulus train is known. In experiments where only the output postsynaptic response is known, this technique is particularly useful. While the machines themselves cannot be interpreted in a physiological way, the information they provide can be used to classify synaptic dynamics and inform the construction of physiological models. The point of the analysis is to gain as much accurate information from experiments in short term synaptic plasticity as possible without imposing the bias of an assumed underlying physical model. To create synthetic data we use a very simple but otherwise complete model of short-term plasticity that incorporates a “memory” effect through the inclusion of calcium build-up and decay. This has roots in a real physiological process (the flooding of calcium into the presynaptic terminal can trigger the release of neurotransmitter), but we are not interested *per se* in creating a biophysically complete model here. The calcium dynamics simply introduces another time scale into the model, one that is physiologically relevant. We wish to explore the effect of this additional time scale on the complexity of the process.

Computational Mechanics is an area of study pioneered by Crutchfield and colleagues in the 1990s, (Crutchfield and Young, 1989; Crutchfield, 1994; Shalizi and Shalizi, 2002). Finding structure in time series with these techniques has been applied in such diverse arenas as layered solids (Varn et al., 2002), Geomagnetism (Clarke et al., 2003), climate modeling (Palmer et al., 2002), financial time series (Park et al., 2007), and more recently ecological models (Boschetti, 2008) and large scale multi-agent simulations (Parikh et al., 2016). In neuroscience, to name a few only, we note one application to spike train data (Haslinger et al., 2013), and a recent publication by Marzen et al. on the time resolution dependence of information measures of spike train data (Marzen et al., 2015).

We employ some of the simplest ideas from this body of work, namely decomposing a discrete time-discrete state data stream into causal states, which are made up of sequences of varying length that all give the same probability of predicting the same next data point in the stream. The data are discrete time by construction, and made into discrete symbols through a partition, so the process can be described by symbolic dynamics. We use the Causal State Splitting Reconstruction (CSSR) algorithm on the data to create the causal states and assemble a HMM that represents the transitions between the states, while emitting a certain symbol. This allows us to classify the synapse types and gives an idea of the differences in the history dependence of the processes as well.

Using an a priori model for short-term synaptic dynamics and fitting it to data, while a perfectly valid approach, allows only for the discovery of the parameters in the model and possibly a necessary model reduction to remove any parameter dependencies (too many parameters in the model for the data set to fit). The alternative approach is to allow the data itself to create the model. From these “data driven” models, conclusions can be drawn about the properties of the synapse that are *explicitly discoverable* from the experimental data. The ultimate goal is a categorization of the types of processes a synapse can create, and an assignment of those to different synapse types under varying conditions. Note that the complexity or level of biophysical detail of our model synapse is not important to this end. In fact, the best way to calibrate this method is using the simplest possible model of the dynamics that captures the history dependence of the plasticity. This is not consonant with the goal of incorporating as many physiological features as possible, whether they affect the dynamics significantly or not. In fact, in most cases the limited data in any electro-physiological experiment precludes identifying more than a handful of parameters in an a priori model, a point we discuss in Stone et al. (2014). Our goal is to classify the sort of filter the synapse creates under certain physiological conditions, rather than to identify specific detailed cellular level mechanisms.

We are motivated in this task by the work of Kohus et al. (2016), in which they present a comprehensive data set describing connectivity and synaptic dynamics of different interneuron (IN) subtypes in CA3 using paired cell recordings. They apply dynamic stimulation protocols to characterize the short-term synaptic plasticity of each synaptic connection across a wide range of presynaptic action potential frequencies. They discovered that while PV+ (parvalbumin positive) cells are depressing, CCK+ (Cholecystokinin positive) INs display a range of synaptic responses (facilitation, depression, mixed) depending upon postsynaptic target and firing rate. Classifying such a wide range of activity succinctly is clearly useful in this context. The discovery that the rate of particular observed oscillations in these cells (called sharp wave ripples) may be paced by the short-term synaptic dynamics of the PV+BC in CA3 demonstrates the importance of these dynamics in explaining complex network phenomena.

The paper is organized as follows. The construct for an experimental paper with section 2 and section 3 is not an immediately obvious partition of our work, but we use it as best we can. In the section 2 we describe the background on the synaptic plasticity model, and some analysis of its properties. We also cover the necessary background from Computational Mechanics. Finally we show how the techniques are explicitly applied to our data. In the section 3 we present the epsilon machines created from data from three types of synapses from our FD model: depressing, facilitating, and mixed, at varying input frequencies. Here we also indicate similarities and differences in the actual machines. In the section 4 we speculate on the reasons for these features by referring back to the original model. In the last section we indicate directions for future work.

## 2. Materials and methods

### 2.1. Model of synaptic plasticity

In Stone et al. (2014), we parameterize a simple model of presynaptic plasticity from work by Lee et al. (2008) with experimental data from cholinergic neuromodulation of GABAergic transmission in the hippocampus. The model is based upon calcium dependent enhancement of probability of release and recovery of signalling resources (For a review of these mechanisms see Khanin et al., 2006). It is one of a long sequence of models developed from 1998 to the present, with notable contributions by Markram et al. (1998) and Dittman et al. (2000). The latter is a good exposition of the model as it pertains to various types of short term plasticity seen in the central nervous system, and the underlying dependence of the plasticity is based on physiologically relevant dynamics of calcium influx and decay within the presynaptic terminal. In our work, we use the Lee model to create a two dimensional discrete dynamical system in variables for calcium concentration in the presynaptic area and the fraction of sites that are ready to release neurotransmitter into the synaptic cleft.

In the rest of this section we outline the model, which is used to generate synthetic data for our study of causal state models, or epsilon machines, of short-term plasticity.

In the model the probability of release (*P*_*rel*_) is the fraction of a pool of synapses that will release a vesicle upon the arrival of an action potential at the terminal. Following the work of Lee et al. (2008), we postulate that *P*_*rel*_ increases monotonically as function of calcium concentration in a sigmoidal fashion to asymptote at some *P*_*max*_. The kinetics of the synaptotagmin-1 receptors that binds the incoming calcium suggests a Hill equation with coefficient 4 for this function. The half-height concentration value, *K*, and *P*_*max*_ are parameters determined from the data.

After releasing vesicles upon stimulation, some portion of the pool of synapses will not be able to release vesicles again if stimulated within some time interval, i.e., they are in a refractory state. This causes “depression;” a monotonic decay of the amplitude of the response upon repeated stimulation. The rate of recovery from the refractory state is thought to depend on the calcium concentration in the presynaptic terminal (Dittman and Regehr, 1998; Wang and Kaczmarek, 1998). The model has a simple monotonic dependence of rate of recovery on calcium concentration, a Hill equation with coefficient of 1, starting at some *k*_*min*_, increasing to *k*_*max*_ asymptotically as the concentration increases, with a half height of *K*_*r*_.

The presynaptic calcium concentration itself, [*Ca*], is assumed to follow first order decay kinetics to a base concentration, [*Ca*]_*base*_. At this point we choose that [*Ca*]_*base*_ = 0, since locally (near the synaptotagmin-1 receptors) the concentration of calcium will be quite low in the absence of an action potential. The evolution equation for [*Ca*] then is simply $\tau_{ca}\frac{d\left[Ca\right]}{dt}=-\left[Ca\right]$ where τ_*ca*_ is the calcium decay time constant, measured in milliseconds. Upon pulse stimulation, presynaptic voltage-gated calcium channels open, and the concentration of calcium at the terminal increases rapidly by an amount δ (measured in μ*m*): [*Ca*] → [*Ca*]+δ at the time of the pulse. Note that calcium build-up is possible over a train of pulses if the decay time is long enough relative to the ISI.

As mentioned above, the probability of release *P*_*rel*_ and the rate of recovery, *k*_*recov*_, depend monotonically on the instantaneous calcium concentration via Hill equations with coefficients of 1 and 4 respectively. Rescaling the calcium concentration by δ = δ_*c*_ in the control case, we define *C* = [*Ca*]/δ_*c*_. Then the equations are $P_{rel}=P_{max}\frac{C^{4}}{C^{4}+K^{4}},$ and $k_{recov}=k_{min}+\Delta k\frac{C}{C+K_{r}}$. The variable *R*_*rel*_ is governed by the ordinary differential equation $\frac{dR_{rel}}{dt}=k_{recov}\left(1-R_{rel}\right),$ which can be solved exactly for *R*_*rel*_(*t*). $R_{rel}\left(t\right)=1-\left(1-R_{0}\right){\left(\frac{C_{0}e^{-t}+K_{r}}{K_{r}+C_{0}}\right)}^{\Delta k}e^{-k_{min}t}$. *P*_*rel*_ is also a function of time as it follows the concentration of calcium after a stimulus.

We used experiments in hippocampus to parameterize this model, as part of an exploration of the frequency dependent effects of neuromodulation. Whole-cell recordings were performed from synaptically connected pairs of neurons in mouse hippocampal slices from PV-GFP mice (Lawrence et al., 2015). The presynaptic neuron was a PV basket cell (BC) and the postsynaptic neuron was a CA1 pyramidal cell. Using short, 1–2 ms duration suprathreshold current steps to evoke action potentials in the PV BC from a resting potential of −60 mV and trains of 25 of action potentials are evoked at 5, 50, and 100 Hz from the presynaptic basket cell. The result in the postsynaptic neuron is the activation of *GABA*_*A*_-mediated inhibitory postsynaptic currents (IPSCs). Upon repetitive stimulation, the amplitude of the synaptically evoked IPSC declines to a steady-state level. These experiments were conducted with 5, 50, and 100 Hz stimulation pulse trains, with and without the neuromodulator muscarine, in order to test frequency dependent short term plasticity effects.

The peak of the measured postsynaptic IPSC is presumed to be proportional to the total number of synapses that receive stimulation *N*_*tot*_, which are also ready to release (*R*_*rel*_), e.g., *N*_*tot*_*R*_*rel*_, multiplied by the probability of release *P*_*rel*_. That is, peak IPSC ~*N*_*tot*_*R*_*rel*_*P*_*rel*_. *P*_*rel*_ and *R*_*rel*_ are both fractions of the total, and thus range between 0 and 1. Without loss of generality, we consider peak IPSC to be proportional to *R*_*rel*_*P*_*rel*_.

From the continuous time functions describing *C*, *R*_*rel*_, and *P*_*rel*_, we constructed a discrete dynamical system (or “map”) that describes *P*_*rel*_*R*_*rel*_ upon repetitive stimulation. Given an ISI of *T*, the calcium concentration after a stimulus is *C*(*T*) + Δ (Δ = δ/δ_*c*_), and the peak IPSC is proportional to *P*_*rel*_(*T*)*R*_*rel*_(*T*), which depend upon *C*. After the release, *R*_*rel*_ is reduced by the fraction of synapses that fired, e.g., *R*_*rel*_ → *R*_*rel*_−*P*_*rel*_*R*_*rel*_ = *R*_*rel*_(1 − *P*_*rel*_). This value is used as the initial condition in the solution to the ODE for *R*_*rel*_(*t*). A two dimensional map (in *C* and *R*_*rel*_) from one peak value to the next is thus constructed. To simplify the formulas we let *P* = *P*_*rel*_ and *R* = *R*_*rel*_. The map is

(1) $$\begin{array}{r} C_{n+1}=C_{n}e^{-T}+\Delta , \end{array}$$

(2) $$\begin{array}{r} P_{n+1}=P_{max}\frac{C_{n+1}^{4}}{C_{n+1}^{4}+K^{4}}, \end{array}$$

(3) $$\begin{array}{r} R_{n+1}=1-\left(1-\left(1-P_{n}\right)R_{n}\right){\left(\frac{C_{n}e^{-T}+K_{r}}{K_{r}+C_{n}}\right)}^{\Delta k}e^{-k_{min}T}. \end{array}$$

Following this notation the peak value upon the *n*th stimulus is *Pr*_*n*_ = *R*_*n*_*P*_*n*_, where *R*_*n*_ is the value of the reserve pool before the release reduces it by the fraction (1 − *P*_*n*_).

Data from the experiments were fitted to the map using the Matlab package *lsqnonlin*, and with Bayesian techniques (Haario et al., 2006). The value of *P*_*max*_ was determined by variance-mean analysis, and is 0.85 for the control data and 0.27 for the muscarine data. The common fitted parameter values for both data sets are shown in Table 1.

**Table 1:** Parameter values.

**Parameter**	**Fitted value**
*K*	0.2
*k*_*min*_	0.0017 1/ms
*k*_*max*_	0.05171/ms
*K*_*r*_	0.1
τ_*ca*_	1.5 ms

For the control data set Δ = 1, and the muscarine data set has the fitted value of Δ = 0.17. From this result it is clear that the size of the spike in calcium during a stimulation event must be much reduced to fit the data from the muscarine experiments. This is in accordance with the idea that mAChR activation reduces calcium ion influx at the terminal.

#### 2.1.1. Analyzing the map

It is common in the experimental literature to classify a synapse as being “depressing” or “facilitating,” depending upon its response to a pulse train at some relevant frequency. Simple models can be built that create each effect individually. The model here combines both mechanisms so that, depending upon the parameters, both facilitation and depression and a mixture of the two can be represented (Lee et al., 2008). Note that facilitation is built into this model through the calcium dependent *P* value and rate of recovery. For instance, by varying the parameters we can create a “mock” facilitating synapse, where the size of the response increases with increasing frequency of input stimulation, or a “mixed” synapse, where the response is depressed for low and high frequency, but increases comparatively for moderate values of the frequency.

We are able to “tune” the parameters in the map from the fitted values to realize these cases, and the results are shown in Table 2. To attain more complicated dynamics we must first increase the calcium decay time to 30 ms, a much larger value that has never-the-less been found in fitting the model to electrophysiological data from other synapses (Lawrence lab, unpublished results). The build-up of calcium means a larger recovery rate, but the probability of release ranges only up to *P*_*max*_ = 0.6, and over a larger concentration range of the calcium (*K* = 4.0 for facilitating and *K* = 1.0 for mixed), off-setting the effect of the larger amount of calcium from the build-up to a varying degree. The competition between increasing probability of release and decreasing *R* creates the local maximum in the mixed case, which is also present in the facilitating case, but for frequencies outside the physiological range.

**Table 2:** Parameter values for “mock” synapses.

**Parameter**	**Facilitating**	**Mixed**
*K*	4.0	1.0
*k*_*min*_	0.002 1/ms	0.002 1/ms
*k*_*max*_	6.0 1/ms	6.0 1/ms
*K*_*r*_	0.1	0.1
τ_*ca*_	30 ms	30 ms

**Figure 1:**
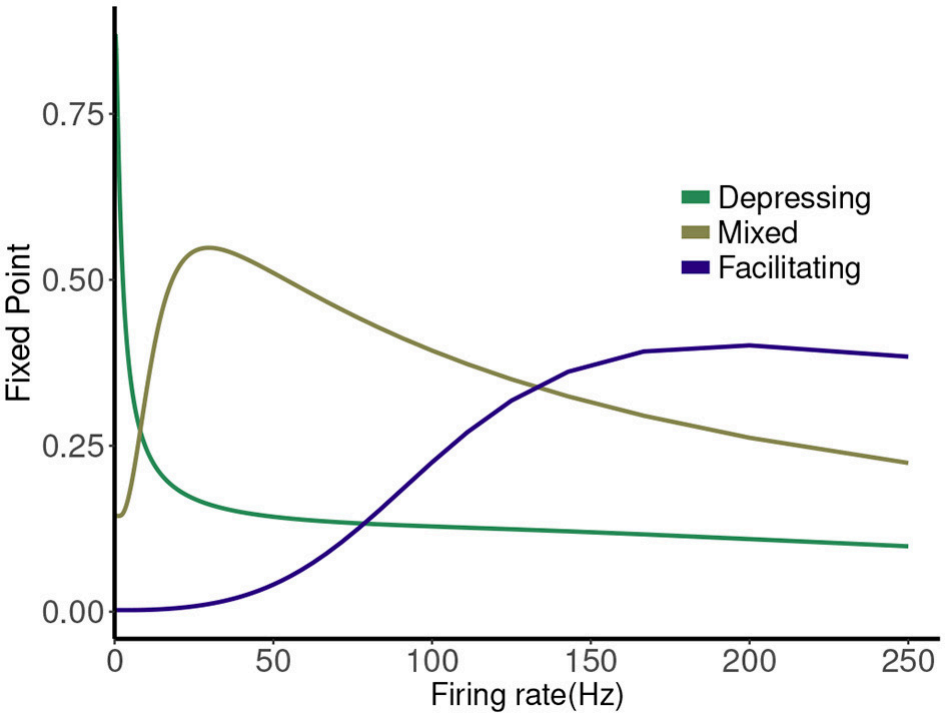
Fixed point values of normalized postsynaptic response for three synapse models of “depressing,” “mixed,” and “facilitating” stimulated by Poisson spike trains with mean firing rates ranging from 0.1 to 250.

The map has a single attracting fixed point, and the collapse to this fixed point from physiological initial conditions is very rapid (Stone et al., 2014). The value of the fixed point depends on the frequency (1/*T*), and plotting this is a good way to represent the different types of synaptic dynamics. In Figure 1 we plot the expression for the fixed point ($\bar{Pr}=\bar{P}\times \bar{R}$ or $\bar{PR}$) of the deterministic map vs. rate for three cases. For instance, the depressing synapse fixed point decreases from *P*_*max*_ (for one stimulus, or zero frequency) monotonically, with a quick decay over 0–10 Hz, and a slower decay to zero following. The facilitating synapse fixed point increases over the physiological range shown, but decreases for larger values of the frequency. The mixed synapse fixed point starts at a base value of 0.3 for one stimulus, increases to a local max near 50 Hz and decays thereafter. The “resonance” indicated by the local maximum gives the mixed synapse more complicated linear filtering properties than the other two in the physiological frequency range.

#### 2.1.2. The depressing synapse

The interplay of the presynaptic probability of release and the rate of the recovery can create a non-linear filter of an incoming stimulus train. To investigate this idea, in Bayat et al. we consider the distribution of values of *Pr* created by exponentially distributed random ISIs for varying rates λ, or mean ISI, denoted < *T* >= 1/λ for the depressing synapse. Doing so explores the filtering properties of the synapse when presented with a Poisson spike train. We also present results from numerical studies to determine of the effect of varying the mean rate of the pulse train. The information processing properties, in the form of mutual information and multivariate mutual information, of the synapse at physiological frequencies are compared. We found that the mutual information peaked around 3 Hz, when the entropy of the *Pr* distribution was at its maximum, for both muscarine and control parameter sets.

We also determined that the random variable describing the calcium concentration has a closed form distribution, and indeed a well-known distribution. However, this is not the case for the variable *R* due to the complexity of the map, and so a closed form for the distribution of *Pr* = *PR* is not possible. However, we can understand it partially by considering the mechanisms involved, and using some information from the deterministic map, namely the expression for the fixed point. If the *Pr* value is directly determined by the fixed point value for the ISI preceding it, we would be able to convert the distribution of the ISIs into that of the *Pr*s by using composition rules for distributions of random variables. We examine this when the calcium decay time (τ_*ca*_) is notably smaller than the ISI (*T*). With this approximation *C, P*, and *R* have time in between pulses to decay to their steady state value before another pulse. This means that the fixed point value for a rate given by 1/*T* where *T* is the preceding interspike interval is more likely to give a good estimate of the actual value or *Pr* = *PR*.

It was shown in Stone et al. (2014) that in this case $\bar{C}\to \Delta$ as *T* increases and hence $\bar{P}\to P_{max}$. Therefore, the fixed point for *R*, $\left(\bar{R}\right)$ is then

$$\bar{R}=\frac{1-e^{-k_{min}T}}{1-\left(1-P_{max}\right)e^{-k_{min}T}}.$$

With this simplification we found the probability density function (PDF) of $\bar{R}$ given an exponential distribution of the variable *T*. For simplicity of notation, we use $X=\bar{R}$ and $Y=\bar{PR}$.

If *X* is a random variable, then an analytic expression for its PDF is given by

(4) $$\begin{array}{r} f\left(x|\lambda ,c,k_{min}\right)=\frac{\lambda \left(1-c\right)}{k_{min}}{\left(1-x\right)}^{-\left(1-\lambda /k_{min}\right)}{\left(1-cx\right)}^{-\left(1+\lambda /k_{min}\right)}, \end{array}$$

where *c* = 1 − *P*_*max*_, λ > 0 is the mean Poisson rate and *k*_*min*_ > 0 is the baseline recovery rate. The distribution is supported on the interval [0, 1]. Similarly, we can compute the analytical expression of the PDF of fixed point *Y*. We will refer to this in what follows as the *stochastic fixed point*. Hence, the PDF for the stochastic fixed point is

(5) $$\begin{array}{l} f\left(y|\lambda ,c,k_{min}\right)=\\ \frac{\lambda P_{max}\left(1-c\right)}{k_{min}}{\left(P_{max}-y\right)}^{-\left(1-\lambda /k_{min}\right)}{\left(P_{max}-cy\right)}^{-\left(1+\lambda /k_{min}\right)}. \end{array}$$

**Figure 2:**
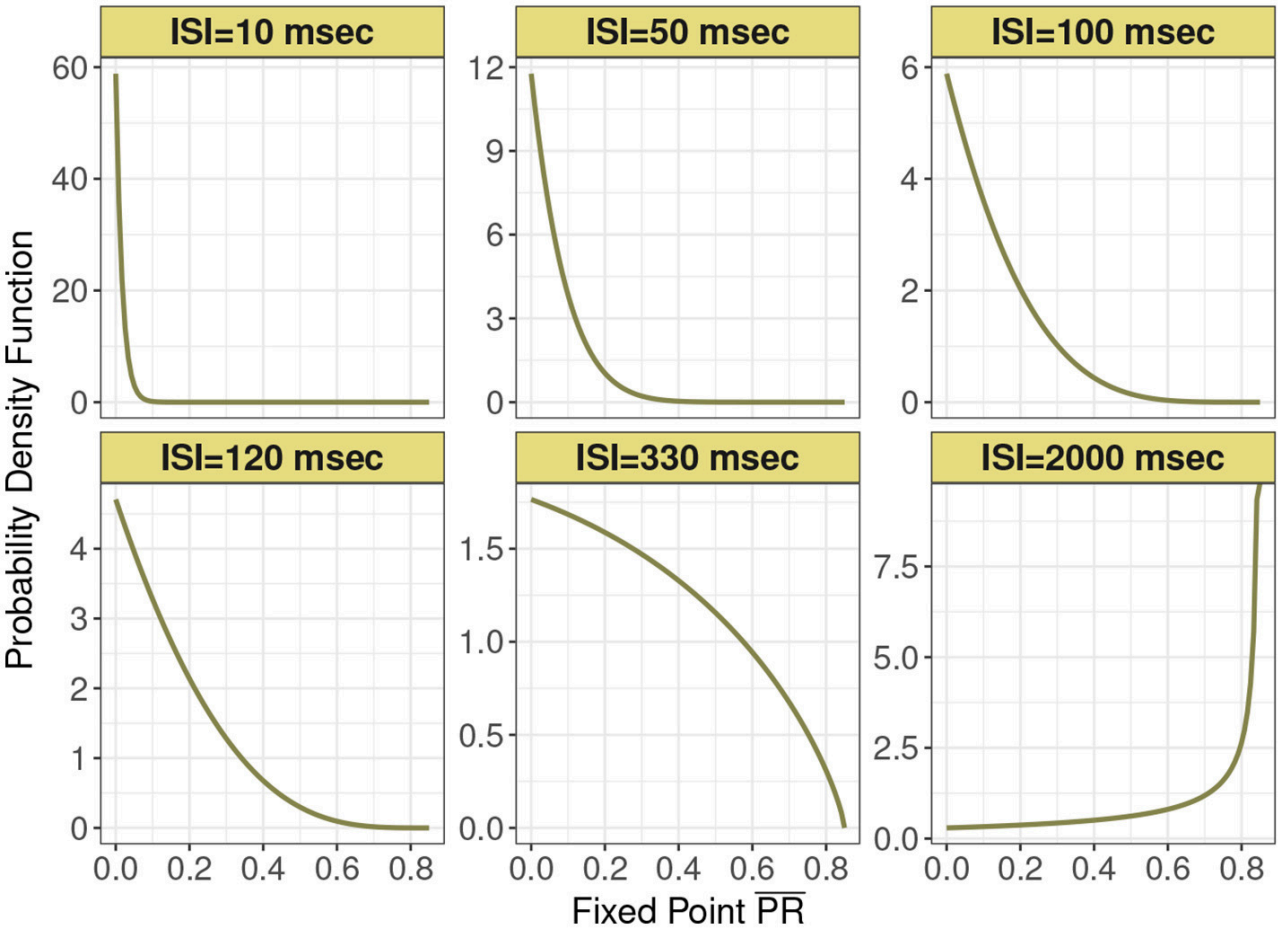
Probability density function of the normalized postsynaptic response fixed point $\bar{PR}$ for six interspike interval variants of 10, 50, 100, 120, 330, and 2,000 ms under analytic expression. Minimum recovery rate *k*_*min*_ is 0.0013 and maximum probability of release *P*_*max*_ is 0.85 under the control condition in depressing synapse model.

This distribution is supported on the interval [0, *P*_*max*_]. Figure 2 shows this expression for different mean input ISI, in milliseconds.

**Figure 3:**
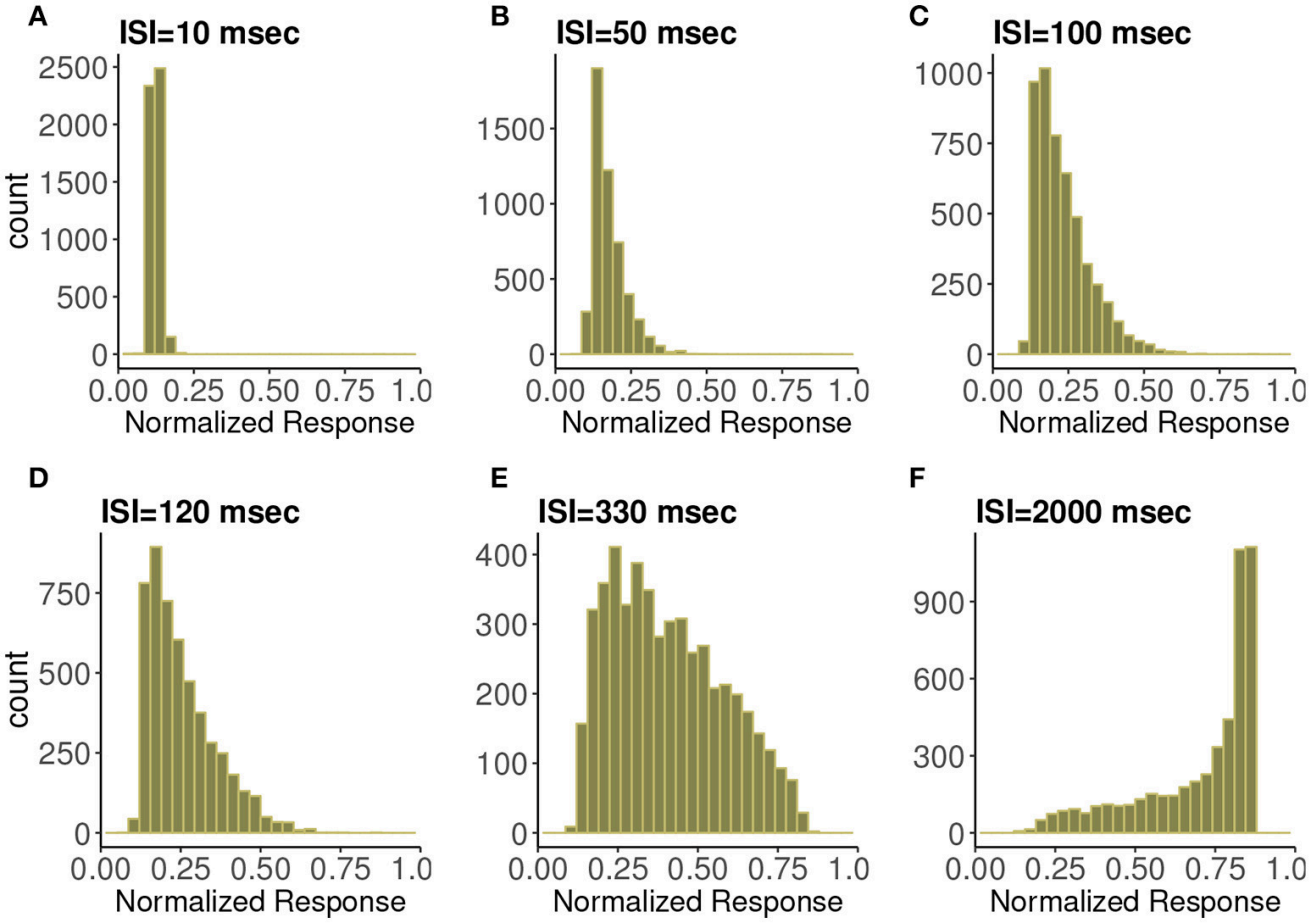
Frequency distributions of normalized postsynaptic response for varying presynaptic interspike interval values of **(A)** 10, **(B)** 50, **(C)** 100, **(D)** 120, **(E)** 330, and **(F)** 2,000 in milliseconds. We consider very small calcium decay time τ_*ca*_ under the control condition. We can observe the similarity with Figure 2 which indicates the agreement with the analytic expression.

In Figure 3 are histograms of *Pr*-values obtained numerically from the map with very small τ_*ca*_, with an exponentially distributed *T* random variable and other parameters from the control set, as in Figure 2. The similarity between the two is evident. Apparently this approximation captures the shape of the distribution and how it changes with varying input spike train rate.

We are now convinced that we understand the primary driver of the variation of the probability distribution of the response to the input mean rate. However, as mentioned before, the creation of a distribution automatically ignores the causality in the sequence of the responses. In the next section we describe a method for assessing this causality directly from the response data.

### 2.2. Computational mechanics background

We can use distribution to compute measures of information transfer between input spike trains and output *Pr*s. However, the question of how far back in a spike train the synapse “remembers,” or, how far back in the spike train is important for predicting the output, is difficult to answer, even using multivariate mutual information measures. Instead we propose a method for describing the process in terms of output only, with the goal of classifying the complexity of the underlying synaptic dynamics. This method relies on the ideas of “computational mechanics” developed by Crutchfield and colleagues in the 1990s.

The material presented in this section is drawn from many of the seminal papers by Crutchfield et al. (Crutchfield and Young, 1989; Crutchfield, 1994; Shalizi and Shalizi, 2002), much of which is quite technical. In what follows we outline the key ideas that we have used in our analysis, but note that the theoretical underpinnings of the ideas are completely described in this body of work and we refer the reader to these papers for more detail.

Imagine a black box experimental system and its measurement channel. Inside the black box is a three state system or process. The measurements are a sequence of symbols (0, 1) generated upon transitions between the unseen states in the black box. The measurement channel itself acts to map the internal state sequence ⋯*BCBAA*⋯ to a measurement sequence of symbols ⋯01110⋯. The black box system is assumed to be Markovian, meaning that the transition probability from one state to another depends only upon the current state. The observed symbol sequence, generated upon transitions between states, make the system a hidden Markov process. From the point of view of the observer, how many of the system's properties be inferred from the observed symbol sequence? Can a model of the hidden process be created from this data stream? Can the model be used to predict the future symbols in the sequence?

Let the symbol sequence be represented by *S*. With information from the past $\overset{⃖}{S}$, we want to make a prediction about the future $\vec{S}$. The formative idea is to find past sequences of measurements (histories) leading to the same future. Once these states are identified, the transitions between them can be inferred from *S*. The states themselves and the transition matrix are called the ϵ-machine for the process. A finite state ϵ-machine is a Unifilar Hidden Markov Model given by $M=\left\{S,\left\{T^{\left(s\right)},s\in A\right\}\right\}$ where unifilar means for each state $\sigma_{i}\in S$ and each symbol x∈X there is at most one outgoing edge from state σ_*i*_ and output symbol *x*.

An ϵ-machine captures the (temporal) patterns in the observations and reflects the causal structure of the process. With this model, we can extrapolate beyond the original observations to predict future behavior of a system. The ϵ-machine is further defined to be the unique, minimal and maximally optimal model of the observed process. It can model stationary stochastic processes with states that represent equivalence classes of histories with no significant difference in their probability distribution over the future events.

#### 2.2.1. Epsilon machine construction

Consider a portion of a contiguous chain of random variables: *X*_*n*:*m*_ = *X*_*n*_*X*_*n*+1_⋯*X*_*m*_, *m* > *n*. A semi-infinite chain is either: *X*_*n*:_ = *X*_*n*_*X*_*n*+1_⋯, which is called the future, or *X*_:*n*_ = ⋯*X*_*n*−2_*X*_*n*−1_, the past. The bi-infinite chain of random variables is denoted *X*_:_. A *process* is specified by the distribution *Prob*(*X*_:_).

Assume the process is stationary and that a realization of length *L* has this property: *Prob*(*X*_1:*L*_) = *Prob*(*X*_*n*:*L*+*n*−1_) for all *n* ∈ *Z*. The values of *X*_*i*_, the *x*_*i*_, are drawn from a finite alphabet, A. In our case we use two symbols, 0 and 1, and a sample finite realization of the process would look like: 00111001001, for instance.

A causal state $\sigma^{+}\in S^{+}$ is a set of pasts grouped by the equivalence relation ~^+^:

$$x_{:0}\sim^{+}x_{:0}^{\prime }<=>Prob\left(X_{0:}|X_{:0}=x_{:0}\right)=Prob\left(X_{0:}|X_{:0}=x_{:0}^{\prime }\right)$$

Two histories are equivalent if and only if they have the same conditional distribution of futures. Groups of specific blocks, e.g., 011, 10, 1011 might all be in the same causal state. At a time *t*, $S_{t}^{+}$ is a random variable drawn from $\sigma^{+}\in S^{+}$ and $\cdots S_{-1}^{+}S_{0}^{+}S_{1}^{+}\cdots S_{t}^{+}$ is a causal state process. Each causal state has a future morph $Prob\left(X_{t:}|\sigma_{t}^{+}\right)$, the conditional measure of futures that can be generated from it. Each state inherits a probability $\pi \left(\sigma_{t}^{+}\right)$ from the processes measure over all pasts *Prob*(*X*_:*t*_). A generative model is constructed out of the causal states by giving the causal state process transitions:

$$T_{\sigma \sigma^{\prime }}^{\left(x\right)}=Prob\left(S_{t+1}^{+}=\sigma^{\prime },X_{t}=x|S_{t}^{+}=\sigma \right)$$

that give the probability of generating the next symbol *x* and while starting from state σ and ending in state σ′. A process' forward-time ϵ-machine is the tuple $\left\{A,S^{+},\left\{T^{\left(x\right)}:x\in A\right\}\right\}$ For a discrete time, discrete alphabet process, the ϵ machine is its minimal unifilar HMM. Minimal means the smallest number of states, and unifilarity means the next state is known given the current state and the next symbol. E.g., the probability of the transition $Prob\left(S_{t+1}^{+}|X_{t}=x,S_{t}^{+}=\sigma \right)$ has support on a single causal state. The *statistical complexity* of an epsilon machine is defined to be the entropy of the causal state distribution, e.g., $H\left[S^{+}\right]$.

The task of creating the epsilon machine is not a simple one and generally is quite computationally intense. There has been much work on creating code for this purpose, and we rely on available software. For instance, in these preliminary results we use the Causal-State Splitting Reconstruction Algorithm (CSSR) (Shalizi and Klinkner, 2004) to create the machine from blocks of length *L* starting with *L* = 1 and increasing up to an appropriate maximum. We note that there packages created by Crutchfield's group that use a Bayesian approach for finding machines, resulting in distributions of possible machines on the level transition probabilities for fixed model topology or for inferring the model topology itself (Travers and Crutchfield, 2011; Strelioff and Crutchfield, 2014).

### 2.3. Distributions

**Figure 4:**
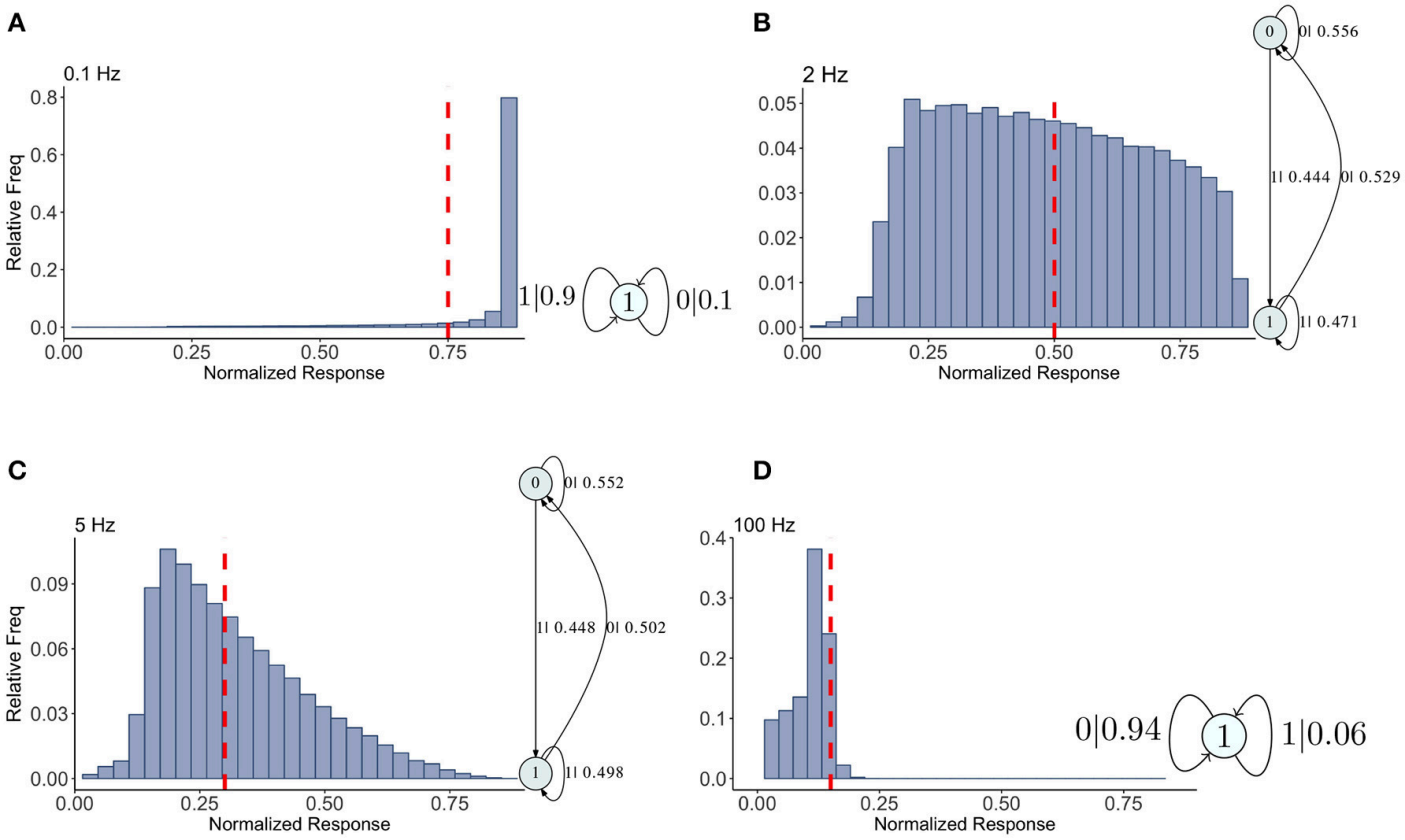
Causal state machines (CSMs) reconstructed and their corresponding relative frequency distributions obtained from depressing FD model. Model is stimulated by Poisson spike trains with mean firing rates **(A)** 0.1, **(B)** 2, **(C)** 5, and **(D)** 100 Hz. The transitions between states are indicated with symbol emitted during the transition (1, large synaptic response; 0, small synaptic response) and the transition probability. In both **(A,D)**, CSMs for 0.1 and 100 Hz Poisson spiking process consist of a single state “1” which transitions back to itself, emitting a large response with probabilities 0.9 and 0.06 for low and very high mean firing rates, respectively. In both **(B,C)**, 2-state CSMs reconstructed for 2 and 5 Hz Poisson spiking process emit large response with nearly similar probabilities.

**Figure 5:**
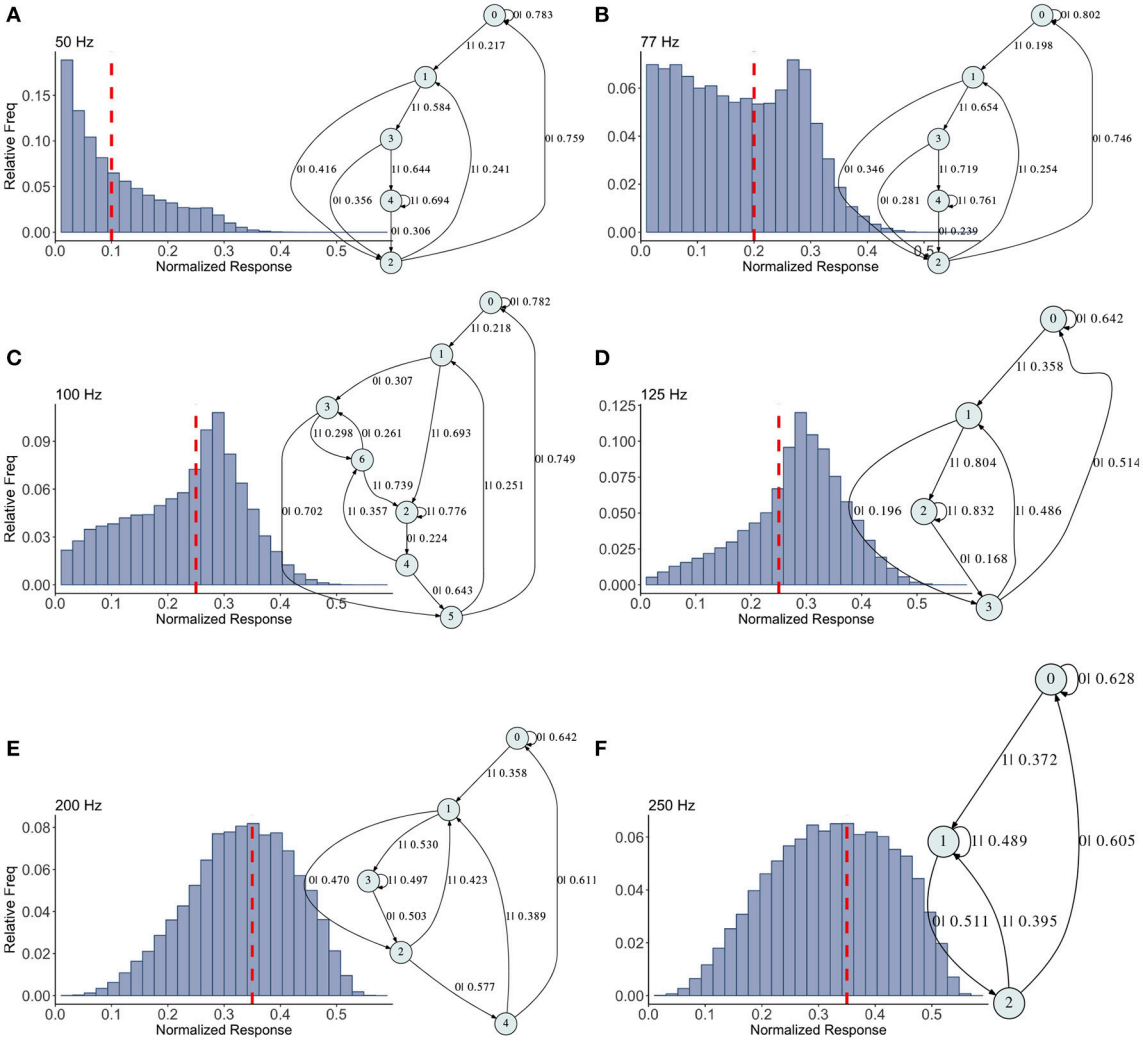
Causal state machines (CSMs) reconstructed and their corresponding relative frequency distributions obtained from facilitating FD model driven by Poisson spike train with mean firing rates **(A)** 50, **(B)** 77, **(C)**100, **(D)** 125, **(E)** 200, and **(F)** 250 Hz. State “0” is the baseline state. Similar graph structure is seen for mean firing rates of 50 and 70 Hz. Under mean firing rate of 100 Hz, the graph structure is more complex with more edges, vertices, and one set of parallel edges from state “3” to “6”. This increase in complexity is somewhat not surprising as this is inflection point where the concavity of the normalized response fixed point for this synapse model changes at this firing rate, (see Figure 1). In non-physiological range from 125 to 250 Hz, the complexity of graph structure decreases.

**Figure 6:**
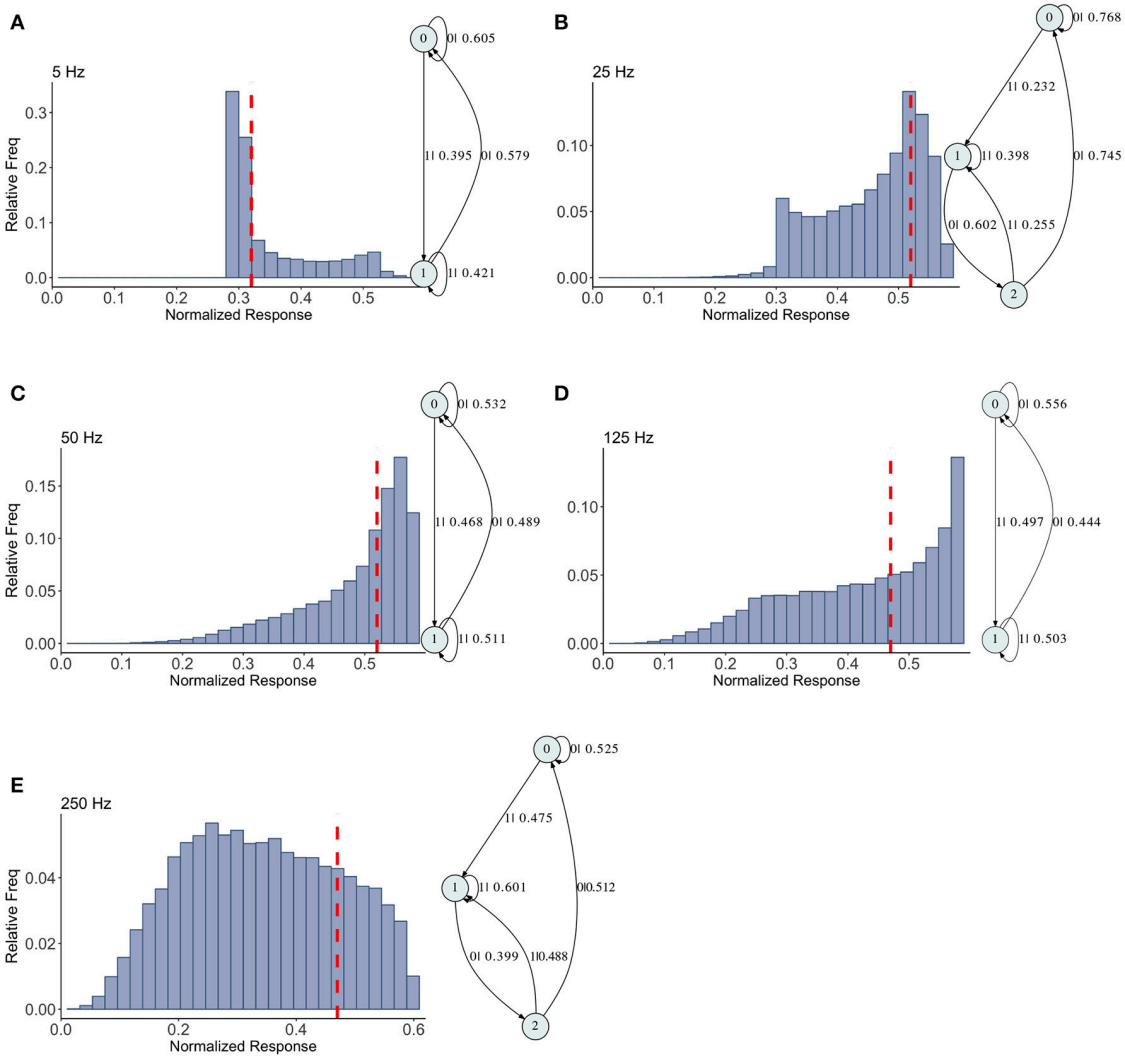
Causal state machines (CSMs) reconstructed and their corresponding relative frequency distributions obtained from mixed FD model driven by a Poisson spike train with mean rates **(A)** 5, **(B)** 25, **(C)** 50, **(D)** 125, and **(E)** 250 Hz. In **(A,C,D)**, CSMs for mean firing rates of 5, 50, and 125 Hz consist of two states with similar structure, emitting successive large responses followed by small responses. 3-State CSM for mean firing rate 25 Hz has more complex graph structure. Note that this is inflection point for this synapse model (see Figure 1).

To create approximations to the distribution of *Pr*-values we computed 2^15^ samples from the stochastic map, after discarding a brief initial transient. The values, ranging between 0 and 1, were placed into evenly spaced bins. The histograms, normalized to be frequency distributions, were computed for a range of mean frequencies (or rates) in the theta range, gamma range, and higher (non-physiological, for comparison). We tested the three different synapse types: depressing, facilitating, and mixed. For parameter values of each, see Tables 1, 2. The histograms themselves are shown in Figures 4–**6**.

In order to create epsilon machines, the *Pr*-values must be partitioned into a sequence of 0's and 1's, which requires the adoption of a threshold value. The choice of this threshold impacts the result, as might be expected. We explore this dependence in Appendix 2, where we show that most of the machines are robust within a finite interval around the chosen threshold. This partition of the output of a real valued map on the interval [0, 1] into a discrete symbol sequence is known as a “symbolic dynamic” and has been studied extensively in dynamical systems theory. For an introductory reference to the mathematical ideas, see Katok's excellent textbook (Katok and Hasselblatt, 1997). If this mapping can be uniquely reversed, the infinite symbol sequence uniquely determines the initial value of the orbit in phase space. This can be proven by finding what is known as a “generating partition” for the iterated map. In the case of the binary shift map, for instance, the partition into two halves of the interval is such a generating partition, because the symbol sequence obtained by following an orbit beginning at *x*_0_ is exactly the binary expansion of *x*_0_. For a general map it is not clear if such a partition exists, or how to find it. The practice is rather to create an equipartition of the phase space (in this case the interval), knowing that as the number of subintervals in the partition increases the accuracy of the representation increases. Here we take a coarse partition, but have limited ourselves to comparing epsilon machines created from symbol sequences from the same partition only to each other, not to any external case. This is similar to the problem of computing the entropy of a distribution with a histogram, which depends explicitly on the number of bins. Finally we note that describing orbits of iterated maps on the unit interval with a symbol sequence by partitioning the interval is common and considered to be generally applicable and advantageous if the iterates are obtained from a numerical simulation or from experimental data. This idea is taken up in Beck and Schögl (1993), and a good introductory textbook on symbolic dynamics for scientists is Lind and Marcus (1995).

### 2.4. Partition

We have considered several options for the thresholding choice. One idea would be to set the threshold at one half *P*_*max*_, differentiating between small and large responses. However, this might obscure some of the more interesting dynamics in the process, so we could make a decision based on the mean, or median of the distribution. Alternatively we can use the fixed point value for the deterministic map, which is close to the mean in low frequency cases. However, if the goal is to uncover as much of the dynamics as possible, we choose the threshold that gives a machine with the maximum statistical complexity. To do so, we computed machines for varying threshold levels in each case, computed the statistical complexity, and took the one with the largest value. We also need to make sure we were not resolving the noise in the process, which guides us to choose a threshold with care if the support of the distribution is quite small, say less than 0.2. This occurs for very low and very high frequencies typically.

See Appendix 2 for an investigation of the effect the partition has upon the resulting machine. For simpler cases finite changes in threshold do not change the topology, only the probabilities. For the facilitating case in mid-range frequencies the machine changes more dramatically as the threshold is varied. Because the statistical complexity measure quantifies the degree of structure present in the data, choosing the machine that maximizes the statistical complexity ensures that it represents the maximum structure present in the data. Then the resulting machines can be compared across the input frequency range.

### 2.5. Machines

After partitioning, the *Pr* time series becomes a sequence of 0's and 1's that can be used to create HMMs. We apply the CSSR algorithm (Shalizi and Klinkner, 2004), using the Matlab package in the Causal State Modeller Toolbox (available online at http://www.mathworks.com/matlabcentral/fileexchange/33217) (Kelly et al., 2012).

CSSR has two user-specified parameters. The significant level α, assigned by χ^2^ or Kolmogorov-Smirnov (KS) tests, determines whether the estimated conditional distribution of histories over the next-symbol is significantly different from all of the state's other morphs. In case of a significant difference, new states are formed for these subsequences. By varying the significance level α, we control the risk of seeing structure that is not there and states merely created due to sampling error, rather than the actual differences between their morphs. Some common choices of α that work well in practice are 0.001, 0.01, 0.1, and 0.05. In our study we set α = 0.01. Also, the CSSR algorithm depends crucially on another user-set parameter, *L*_*max*_, which is the maximum subsequence length considered when inferring the model structure. It is important to find the correct value of *L*_*max*_ as it defines the exponent of the algorithm complexity. Setting *L*_*max*_ too large results in data shortage for long strings, the algorithm tends to produce too many states and hence the results become unreliable. On the other hand, if *L*_*max*_ is too small, the algorithm won't be able to capture all statistical dependencies in the data and the state structure of the inferred machine may not be useful. Finding an optimal choice of *L*_*max*_ is not straight forward. Here we determine the history length according to the relationship derived from Hanson (1993). Based on this formula, for a given number of data points *N*, and fixed significance level α, we choose the maximum length of subsequence *L* such that

$$\sqrt{\frac{{\left|A\right|}^{L_{max}}}{N-L_{max}}}=\alpha .$$

where A is the alphabet size. For instance, for *N* = 10^5^ and α = 0.01 this formula gives *L*_*max*_ = 3 as a starting value. Sometimes it is still too large and another check on *L*_*max*_ is whether every state in the resulting machine contains at least one sequence of that length. If not, the machine is not valid and *L*_*max*_ should be decreased. For a discussion of this see (Shalizi et al., 2002). Here we have two-symbol alphabet A={0,1}, and we use *L*_*max*_ = 3 and α = 0.01.

For cases with less complex dynamics the machine can be resolved with *L* = 2 (maximum of 4 causal states possible), and increasing to *L* = 3 gives the same result. For the more complex cases *L* = 3 (maximum of 8 causal states possible) was needed to capture the dynamics. In each case we checked that the machines had converged in the sense that they did not change significantly when larger data sets are considered. We also checked that the machines were well-conceived using Shalizi's rule of thumb above.

We show machines for the three different types of synapse next. What we find gives us confidence in the both the algorithm for constructing the machine, and the machine itself as a representative of the dynamics. Furthermore, we are able to use these to illustrate some of the pitfalls in relying only on histograms to elucidate the underlying dynamics of the stochastic process.

## 3. Results

Results for the depressing synapse are shown in Figure 4 and details for all these machines can be found in Appendix 1. We indicate on the histograms where the maximum statistical complexity is with a red line. For low frequencies, the probability of getting a large *Pr* value (or a “1”) is quite large, and its epsilon machine captures that dynamic with one state. Similarly for high frequencies the probability of getting a small *Pr* value (or a “0”) is quite large and a one state machine results with the probabilities reversed. For intermediate frequencies, near the maximum entropy value of 2–3 Hz, the epsilon machine has 2 states, indicating a more complicated sequence of low and high *Pr*-values. Both 2 and 5 Hz have identical machines in structure with slight variations in the transition probabilities.

The words in each causal state indicate the kind of sequences that are typical of the synapse. For 2 and 5 Hz, state 0 contains the sequences 00, 10, 000, 010, 100, and 110. State 1 contains 01, 11, 001, 011, 101, and 111. Between the two, all possible sequences of length 2 and 3 are represented. The probability of getting a 0 or a 1 is more or less equally likely from both states. State 0 contains more zeros overall, so it is the lower *Pr* state. Note that the transition from state 0 to state 1 occurs with the emission of a 1, so the occurrence of a 1 in the sequence drives the dynamic to state 1, and visa versa. This is a kind of sorting of sequences into words with more zeros and those with more 1's. There is nothing particularly “hidden” in this HMM. For us it means the dynamics of the synapse are best understood in terms of the histograms. There is nothing particularly complex in the filter produced by the map.

We have already seen that the histograms for the depressing synapse are well represented by the stochastic fixed point distribution. And even though the distribution sloshes around as the frequency is varied, there is little change in complexity in the epsilon machines through this range. There are several ways to interpret this result. One is that the very short τ_*ca*_ means there is little correlation in calcium time series, which in turn determines the correlation in *P* and, indirectly and directly, *R*. We examine this idea further in section 4. Another way is to consider the histograms themselves which are either fairly flat, or with a single peak at smaller *Pr*-values and an exponential type tail to the right. The structure is simple, and can be understood as a “stochastic fixed point” filter of the incoming Poisson spike train. All this is in contrast with the results for the facilitating synapse, which we show in Figure 5.

Histograms of the output *Pr* are shown in Figures 5A–F, for 50, 77, 100, 125, 200, 250 Hz, respectively, along with their corresponding epsilon machines of *L* = 3. For frequencies less than 50 Hz the machine has one state. Starting at ν = 50, all the machines can be described by referring to a persistent “inner cycle” and “outer cycle.” With the exception of the 100 Hz machine, which has a third cycle, they can be related to one another by graph operations as the frequency is varied. For instance, at 50 and 77 Hz, the machines are topologically similar, with small variations in the transition probabilities. Note however that the histograms are not similar in any obvious way; the epsilon machine identifies the underlying unifying stochastic process. The outer cycle connects state 0 to 1 to 2 and back to 0. The inner cycle connects states 1 to 3 to 4 to 2 and back to 1. An additional transition exists between state 3 and 2, bi-passing state 4. State 4 is notable for its self-connecting edge that emits a “1.” This state also appears in all the other machines. The machine found at 125 Hz is very similar to these: the outer cycle is preserved, though now it connects states 0 to 1 to 3 and back to 0. The inner cycle can be derived from the inner cycle in the lower frequency machines by removing state 3, and sharing an edge with the outer cycle, the one connecting states 1 to 3.

The 200 Hz machine has the same inner cycle as the 125 Hz machine (connecting states 1 to 3 to 2 and back to 1, with a shared edge with the outer cycle from state 1 to state 2). The outer cycle can be made from the 125 Hz outer cycle with the addition of a state between 1 and 3 in that graph, and another edge from the new state back to 1. The machine for 250 Hz is the simplest, and can be derived from the machine at 125 Hz by merging state 1 and 2.

This leaves the most complicated structure, at 100 Hz, with 7 states. However, note that there is still an outer cycle from states 0 to 1 to 3 to 5 and back to 0. The inner cycle connects states 1 to 2 to 4 to 5 and back to 1. The third cycle runs from states 2 to 4 to 6 and back, connecting with the outer cycle at state 3. This connection gives the process another path back to state 0. The point of this rather tedious exercise is to see there is indeed an underlying structure to the overall dynamics of the synapse, with more states and transitions being revealed as the frequency is increased through 100 Hz.

The mixed synapse dynamics are surprisingly less complex, see Figure 6. We set the parameters of the map so that the fixed point spectrum has a local max at physiological frequencies, but apparently this can occur without creating much structure in the histograms, or complexity in the dynamics. The machines at 5, 50, and 125 Hz have 2 states and are the same as the machines at 2 and 5 Hz in the depressing case, with small variation in the transition probabilities. At 25 and 250 Hz the machines are topologically similar with different probabilities on the transitions. They have three states, and state 1 is the same as state 1 in the two state machines. States 0 and 2 together contain the sequences in state 0 of the two state machines. To move from the two state machine to the three state machine another state is added between state 1 and 0 (on that edge) and also linked back to state 1. The machine is also topologically similar to the 250 Hz machine for the facilitating synapse, though the causal states are created with *L* = 2 in the facilitating case.

**Figure 7:**
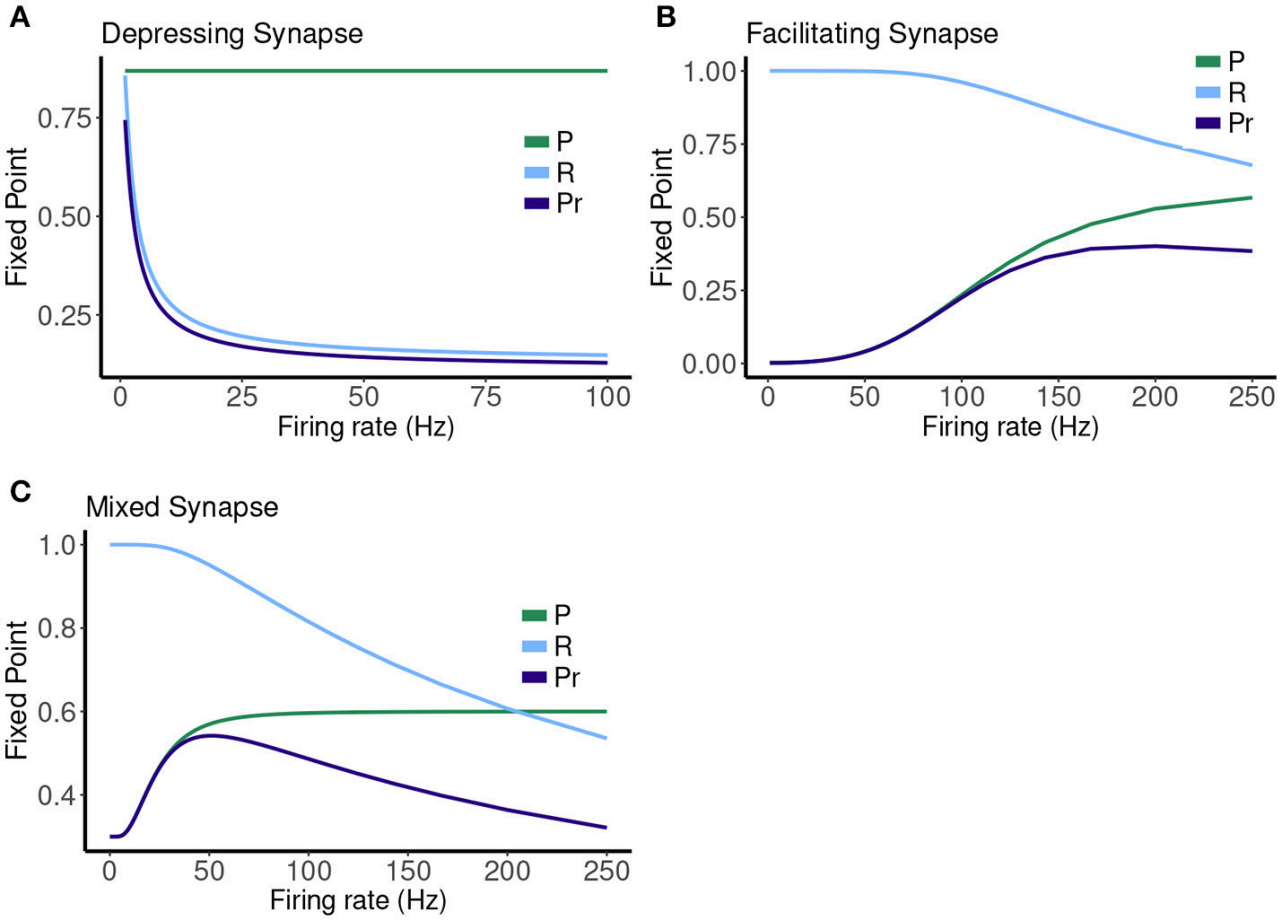
Fixed point values for release probability *P*, fraction of readily releasable pool *R* and normalized postsynaptic response *Pr* for varying mean firing rates ranges from 0.1 to 100 Hz for **(A)** depressing synapse and from 0.1 to 250 Hz for **(B)** facilitating and **(C)** mixed synapse.

The hierarchy of the machines for each set of parameter values is evident, and it is possible to visualize transformations of one machine into another as the firing rate is changed. To sum up these results we plot the statistical complexity of the machines as a function of frequency in each case. See Figure 7. We now seek to connect this back to properties of the synapse model itself.

## 4. Discussion: interpretation of results

The depressing synapse is the simplest of the three cases, and through this investigation it is clear that the formulation of the distribution of the *Pr* in terms of the “stochastic fixed point” gives an almost entire description of the dynamics. For very small and very large frequencies the data points are almost all 1's or 0's, respectively, so the machine has one state. In the small frequency range where the distribution slides from being concentrated at *P*_*max*_ to be concentrated at zero, the epsilon machine shows that the dynamics are still simple, and can be explained by two causal states, one with mostly 0's and the other with mostly 1's. Changing frequency affects the transition probabilities on the edges only.

**Figure 8:**
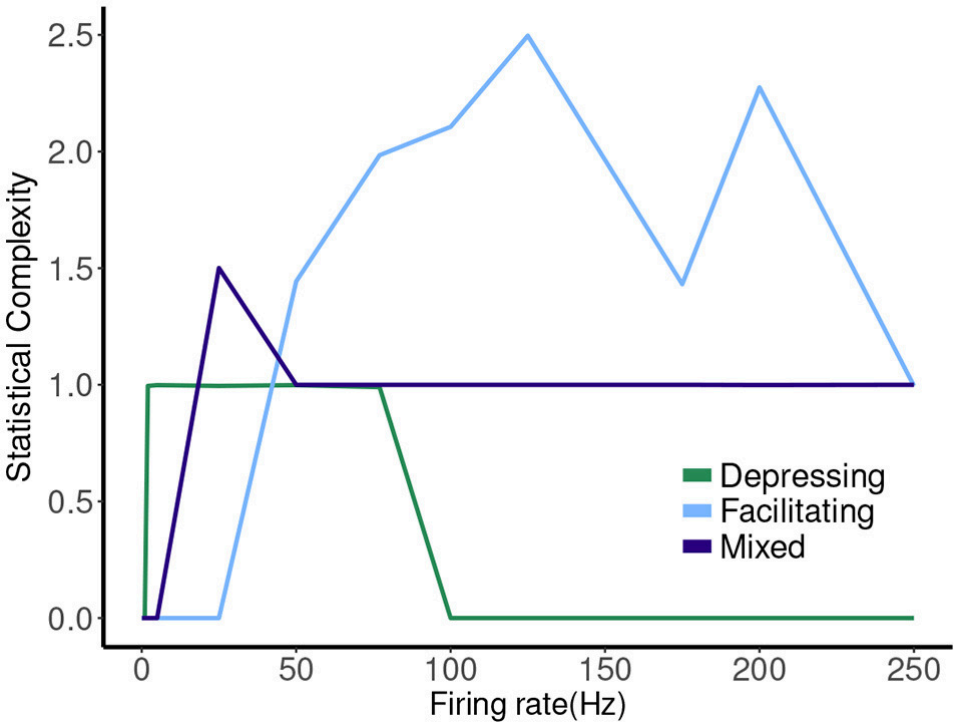
Statistical complexity values obtained from average amount of information of the distribution over causal states as a function of mean firing rates for synapse models, “depressing,” “facilitating,” and “mixed”.

The other two cases are much less simple. More complicated dynamics are possible as the input firing changes. The complexity of the machines for the facilitating synapse compared to the depressing and mixed synapse can be understood by comparing the “decomposed” fixed point spectrum. See Figure 8. Plotting the fixed points in *R* and *P* along with *Pr* shows a striking difference between the three cases. The depressing synapse *Pr* fixed point is entirely controlled by the variation in *R*, as *P* remains constant over the range of frequencies, and the *P* variation happens in a very small range of frequencies near zero. The facilitating synapse has a range of *R*-values across the spectrum, as well as a range of *P*-values. The mixed synapse has a very little variation in *P*. The more complicated machines in both cases occur at frequencies where there is the largest variation in both. Obviously, having a range of response in both *P* and *R* creates the complexity of the machines, however indirectly.

Another way to view this difference is through the calcium decay time. For the depressing synapse τ_*ca*_ is very short, and there is very little correlation in the calcium time series in all but very high frequencies (which are not physiological). The synapse simply filters the Poisson spike train process. In the mixed case, while the calcium time series is more correlated, the lack of variation of the *P* response flattens out any downstream effect on *Pr*. The facilitating case is really in the “goldilocks zone” where the correlation in the calcium time series *can* effect *Pr* through the variation in *P*. A synapse might be expected to be a more complex filter if there is a hidden time dependent variable, such as calcium, that links the two processes of facilitation and depression, as it does in this model for larger τ_*ca*_ or higher frequencies. The exact details of the relationship between the probability of release, and the rate of recovery of R as they depend upon *C* must line up to produce sensitivity in the fixed point values for each in the same frequency range.

Finally, we note that the histograms themselves, from which many information measures are constructed, do not tell the whole story. There is a much more complicated dynamic occurring in the facilitating synapse than the depressing synapse, though comparing the histograms themselves in the two cases does not suggest this. We have also seen the converse, where the machines are the same, even though the distributions are quite different. This implies that both are needed to have a full understanding of such a stochastic process.

## 5. Conclusions

In this paper we demonstrate the validity of using causal state models to more completely describe stochastic short-term synaptic plasticity. These models rely only upon output data from a synaptic connection, knowledge of the input stimulus stream is not required. This will expand the arena of experiments where data can directly inform models, and more importantly uses the data itself to create models. While these models are not physiologically motivated per se, we have shown how we can connect the structure of the model to complexity of the mechanisms involved, a useful first step in a more complete categorization of short term plasticity. Interpreting synaptic plasticity in the language of computation could also be exploited in the construction of large scale models of neural processes involving many thousands of neural connections, and potentially lead to a more complete theoretical description of the computations possible.

Our results also draw direct connections between the causal state models and the deterministic dynamics of the underlying model used to create the data. Specifically, they point to the importance of having variability in both probability of release and the recovery rate of resources with frequency in creating a more complex synaptic filter. This finding can be reversed (at some peril, we realize) to imply that a more complicated machine results from a synapse with such variability. This in turn could be used to inform the development of physiologically accurate models, or direct future experimental design. Interested reader may receive any/all of the code use to create these results by contacting Elham Bayat-Mohktari.

## 6. Future work

The model of the synapse we used to create the data was parameterized from experimental data from an actual depressing synapse in the hippocampus. The experiments gave the synapse uniformly spaced stimuli at fixed frequencies. Our work suggests that a more comprehensive understanding of the dynamics of the synapse could be found by using a predetermined stochastic input, such as a Poisson spike train. The distributions of the responses could then be fit if the desire was to estimate parameters of an a priori model. This fitting could be done using Bayesian techniques as well as standard statistical methods.

The other approach would be to let the data from such an experiment create the model itself, in the form of epsilon machines or perhaps some other form of HMM. We have seen here that the machine reconstruction process can be used for classification purposes, and can uncover features not obvious from the distributions of the response. It is also possible to describe such short term synaptic plasticity as a simple computing operation, or Turing Machine (Copeland, 2004) but the graph model of this is not unifilar, so making a simple connection between it and epsilon machines, or creating a non-unifilar HMMs from data, are topics for further investigation.

Finally, describing the evolution of one epsilon machine to another as a parameter is varied in terms of graph operations could give one more description of an entire range of behavior of a short term plasticity filter, as a parameter is varied. We are currently investigating this approach.

## Author contributions

EB did all the statistical and computational mechanics analyses in the study; JL carried out all experiments and preliminary data analysis; ES conceived of the study, developed the design, and analyzed the results. All authors read and approved the final manuscript.

### Conflict of interest statement

The authors declare that the research was conducted in the absence of any commercial or financial relationships that could be construed as a potential conflict of interest.

## Abbreviations

BC: basket cell
CA1: Cornu Ammonis, early name for hippocampus
FD: facilitation and depression
IPSC: inhibitory postsynaptic current
ISI: interspike interval
KL: Kozachenko and Leonenko
KSG: Kraskov, Stögbauer, and Grassberger
mAChR: muscarinic acetylcholine receptors
MCMC: Monte Carlo Markov Chain
MI: Mutual Information
NT: neurotransmitter
PSR: postsynaptic response
PV: parvalbumin-positive
HMM: Hidden Markov Model
CSSR: Causal State Splitting Reconstruction.
