# Supplementary material for: Data Driven Models of Short-Term Synaptic Plasticity
Source: Front Comput Neurosci. 2018 May 22;12:32. doi: 10.3389/fncom.2018.00032 (PMC5972196; doi:10.3389/fncom.2018.00032)
Supplement: Supplementary file 1 [file Data_Sheet_1.pdf]

Supplementary Material: Data Driven Models of Short-Term Synaptic Plasticity

Elham Bayat Mokhtari, J. Josh Lawrence and Emily F. Stone*
*Correspondence:
Author Name: Emily F. Stone
stone@mso.umt.edu

1 APPENDIX 1

Table S1, S2 and S3 shows the histories of length $L = 2$ or $L = 3$ of the causal states for mixed, facilitating and depressing synapse for different firing rates.

Table S1. The casual states machine reconstructed from mixed synapse data

	State name	Histories $\overleftarrow{x}$	Morph Pr$(1\mid\overleftarrow{x})$	Pr(State)
rate=5 hz	0	00, 10, 000, 010, 100, 110	0.39497	0.59461
	1	01, 11, 001,011, 101, 111	0.42066	0.40538
rate=25 hz	0	00, 000, 100	0.23205	0.54651
	1	01, 11, 011, 001, 111, 101	0.39858	0.28318
	2	10, 010, 110	0.25531	0.17030
rate=50 hz	0	00, 10, 010, 000, 110, 100	0.46753	0.51145
	1	01, 11, 011, 001, 111, 101	0.51053	0.48855
rate=125 hz	0	11, 01, 111, 101, 001, 011	0.50224	0.47144
	1	10, 00, 110, 100, 000, 010	0.44397	0.52855
rate=250 hz	0	00, 000, 100	0.474059	0.54651
	1	01, 11, 011, 001, 111, 101	0.601587	0.28318
	2	10, 010, 110	0.488314	0.17030

Table S2. The casual states machine reconstructed from facilitating synapse data

	State name	Histories $\overleftarrow{x}$	Morph Pr(1|$\overleftarrow{x}$)	Pr(State)
	0	00, 000, 100	0.21658	0.47924
	1	01, 001, 101	0.58359	0.13662
rate=50 hz	2	10, 010, 110	0.24030	0.13662
	3	1, 011	0.64431	0.07973
	4	11, 111	0.69382	0.16778
	0	00, 000, 100	0.19802	0.44910
	1	01, 001, 101	0.65377	0.11920
rate=77 hz	2	10, 010, 110	0.25396	0.11920
	3	1, 011	0.71936	0.07793
	4	11, 111	0.76098	0.23455
	0	000	0.21851	0.27980
	1	001	0.69264	0.08160
rate=100 hz	2	11, 011, 111	0.77652	0.39156
	3	010	0.29741	0.03601
	4	110	0.35668	0.08750
	5	100	0.25076	0.08159
	6	101	0.73926	0.04192
	0	00	0.35821	0.17470
	1	01	0.80351	0.12185
rate=125 hz	2	11	0.83166	0.58160
	3	10	0.48645	0.12184
	0	00, 000	0.35822	0.21312
	1	01, 001, 101	0.52965	0.21672
rate=200 hz	2	10, 010, 110	0.42328	0.21672
	3	11, 011, 111	0.49759	0.22846
	4	100	0.38921	0.12498
	0	0, 00	0.37234	0.35489
rate=250 hz	1	1, 01, 11	0.48872	0.42686
	2	10	0.39453	0.21824

Table S3. The casual states machine reconstructed from depressing synapse data

	State name	Histories $\overleftarrow{x}$	Morph Pr$(1\|\overleftarrow{x})$	Pr(State)
rate= 0.1 hz	0	11, 10, 01, 00, 111, 110, 101, 100, 011, 010, 001, 000	0.902427	1
rate= 2 hz	0	00, 10, 000, 010, 100, 110	0.443366	0.544131
	1	01, 11, 001, 011, 101, 111	0.470802	0.455869
rate= 5 hz	0	11, 01, 111, 101, 001, 011	0.498163	0.471689
	1	10, 00, 110, 100, 000, 010	0.448049	0.528311
rate= 100 hz	0	10, 11, 00, 01, 100, 101, 110, 111, 000, 001, 010, 011	0.0582917	1

APPENDIX 2

Effect of varying partition on CSMs

To establish the validity of the causal state machines created by the CSSR, we consider the effect of varying the threshold in the two state partition of the interval $[0, 1]$. For each of the three parameter sets, at 4 different input frequencies, we create machines from data partitioned by the maximum statistical complexity threshold value, plus or minus 5% of the support of the distribution, the interval $[0, 1]$. In only one instance did this change the topology of the machine. In this case the results raise a red flag, warning that the method needs to be refined in some way. The rest vary only by their statistical properties: state probabilities and transition probabilities. These results are summarized in Tables S4, S5 and S6 and Figures S1, S2 and S3. The facilitating case, which shows the most interesting dynamics of three, is not only the most sensitive to thresholding value, but also to the number of sampled data points at the non-physiological firing rates 100, 200, and 250 Hz. Because of this, it makes even more sense to choose a threshold based on the creating the machine that represents the most structure in the data, i.e. the maximum statistical complexity. Finally, by finding this threshold, one is led naturally to examine the sensitivity of the result to the threshold value.

Table S4. Depressing synapse parameter set.Topology of the machines (number of states) and statistical complexity (SC) as threshold is varied up and down by 0.05 from the maximum statistical complexity value for four different input frequencies.

Firing rate (Hz) / Threshold (τ)/ Number of States/(SC)	0.1	2	5	100
τ/number of states/(SC)	0.75/1/(0)	0.5/2(0.996)	0.3/2/(0.998)	0.15/1/(0)
$\tau + 0.05$/number of states	0.8/1/(0)	0.55/2/(0.960)	0.35/2/(0.945)	0.2/1/(0)
$\tau - 0.05$/number of states	0.7/1/(0)	0.45/2/(0.995)	0.25/2/(0.965)	0.1/1/(0)

Table S5. Mixed synapse parameter set. Topology of the machines (number of states) and statistical complexity as threshold is varied up and down by 0.05 from the maximum statistical complexity value for four different input frequencies.

Firing rate (Hz) / Threshold (τ)/ Number of States/(SC)	0.1	2	5	100
τ/number of states/(SC)	0.32/2/(0.97)	0.52/3/(1.491)	0.52/2/(0.998)	0.47/2/(0.971)
$\tau + 0.05$/number of states	0.37/2/(0.85)	0.57/3/(1.091)	0.57/2/(0.934)	0.52/2/(0.670)
$\tau - 0.05$/number of states	0.27/2/(0.88)	0.47/3/(1.202)	0.47/2/(0.950)	0.42/2/(0.866)

Table S6. Facilitating synapse parameter set. Topology of the machines (number of states) and statistical complexity as threshold is varied up and down by 0.05 from the maximum statistical complexity value for four different input frequencies.

Firing rate (Hz) / Threshold (τ)/ Number of States/(SC)	0.1	2	5	100
τ/number of states/(SC)	0.1/5/(1.470)	0.2/5/(2.02)	0.25/7/(2.106)	0.25/4/(2.49)
$\tau + 0.05$/number of states	0.15/5/(1.410)	0.25/5/(1.184)	0.3/5/(1.711)	0.3/4/(2.266)
$\tau - 0.05$/number of states	0.05/5/(1.452)	0.15/5/(2.001)	0.2/5/(2.056)	0.2/4/(2.451)

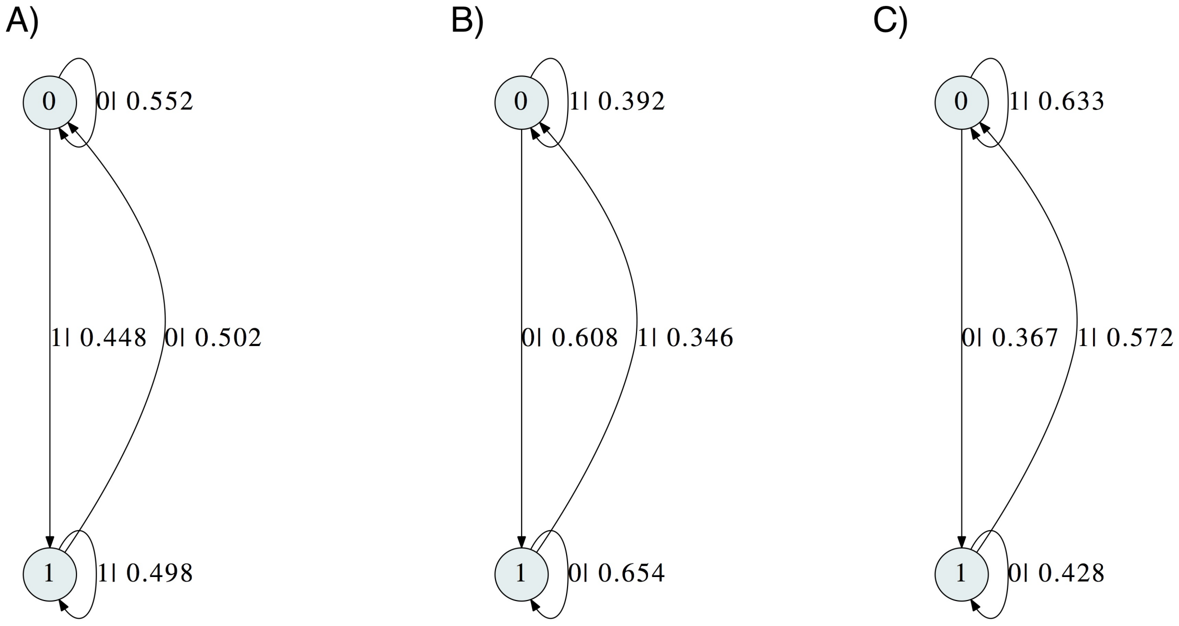

Figure S1 Depressing synapse parameter set. Causal state machines at input frequency of 5 Hz for varying partition threshold. The maximum statistical complexity threshold value, τ, is 0.3 (machine shown in A)), In B) and C) the machine for $\tau + 0.05 = 0.35$ and $\tau - 0.05 = 0.25$, respectively.

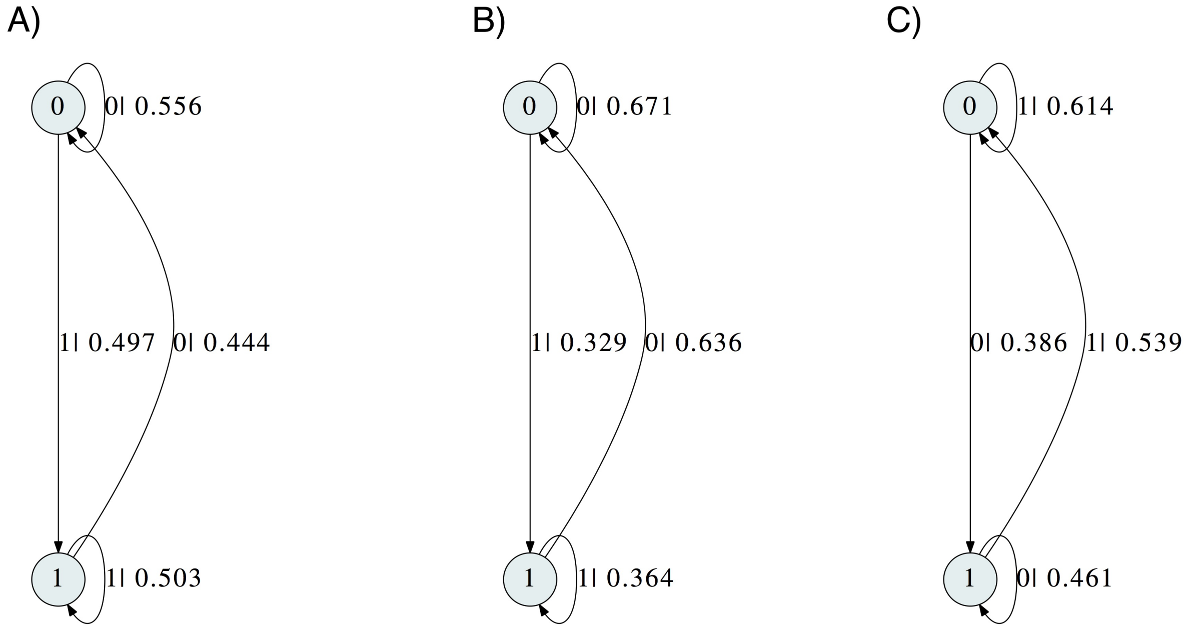

Figure S2 Mixed synapse parameter set. Causal state machines at input frequency of 25 Hz for varying partition threshold. The maximum statistical complexity threshold value, τ, is 0.52 (machine shown in A)), In B) and C) the machine for $\tau + 0.05 = 0.57$ and $\tau - 0.05 = 0.47$, respectively.

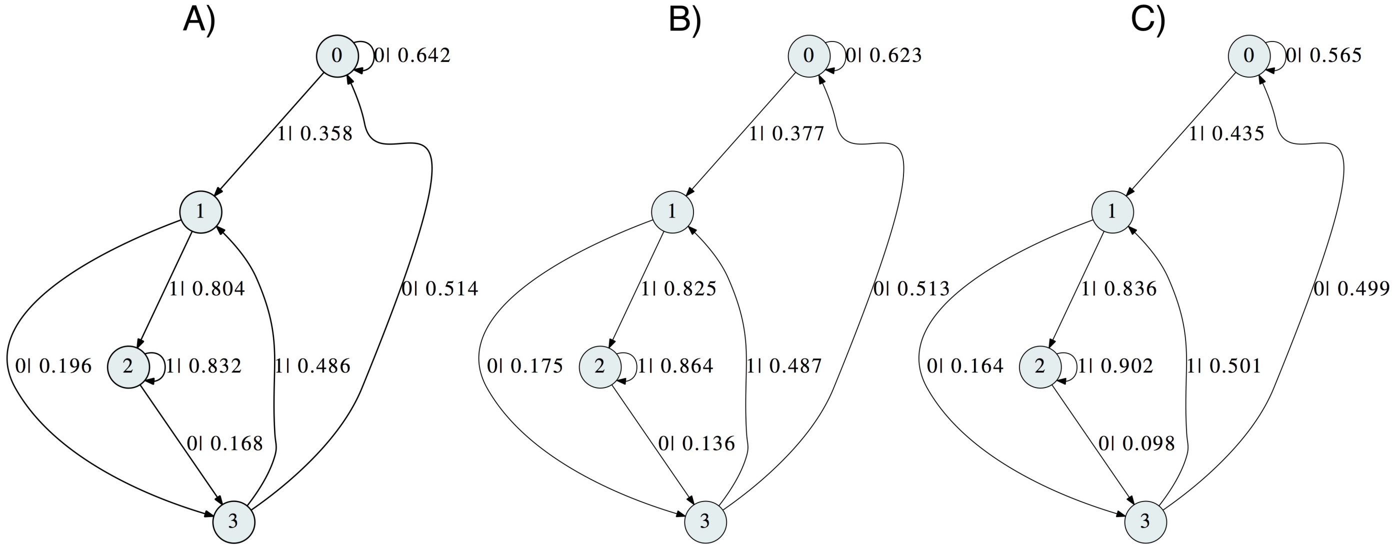

Figure S3 Facilitating synapse parameter set. Causal state machines at input frequency of 125 Hz for varying partition threshold. The maximum statistical complexity threshold value, τ, is 0.25 (machine shown in A)), In B) and C) the machine for $\tau + 0.05 = 0.3$ and $\tau - 0.05 = 0.2$, respectively.
